# 3D NiO hollow sphere/reduced graphene oxide composite for high-performance glucose biosensor

**DOI:** 10.1038/s41598-017-05528-1

**Published:** 2017-07-12

**Authors:** Wei Huang, Shujiang Ding, Yong Chen, Wanjun Hao, Xiaoyong Lai, Juan Peng, Jinchun Tu, Yang Cao, Xiaotian Li

**Affiliations:** 1 State Key Laboratory of Marine Resource Utilization in South China Sea, Key Laboratory of Tropical Biological Resources of Ministry of Education Hainan University, Haikou, 570228 P. R. China; 2 Department of Applied Chemistry, School of Science, State Key Laboratory for Mechanical Behavior of Materials and MOE Key Laboratoryfor Nonequilibrium Synthesis and Modulation of Condensed Matter, Xi’an Jiaotong University, Xi’an, 710049 P. R. China; 3 Laboratory Cultivation Base of Natural Gas Conversion, School of Chemistry and Chemical Engineering, Ningxia University, Yinchuan, 750021 P. R. China; 4 Key Laboratory of Automobile Materials of Ministry of Education, School of Material Science and Engineering, Jilin University, Changchun, 130012 P.R. China

## Abstract

The 3D NiO hollow sphere/reduced graphene oxide (rGO) composite was synthesized according to the coordinating etching and precipitating process by using Cu_2_O nanosphere/graphene oxide (GO) composite as template. The morphology, structure, and composition of the materials were characterized by SEM, TEM, HRTEM, XPS, and Raman spectra, and the electrochemical properties were studied by cyclic voltammetry (CV), electrochemical impedance spectroscopy (EIS), and amperometry. Moreover, the electrochemical activity of the composite materials with different morphologies were also investigated, which indicating a better combination of the NiO hollow sphere and the rGO. Used as glucose sensing material, the 3D NiO hollow sphere/rGO composite modified electrode exhibits high sensitivity of ~2.04 mA mM^−1^ cm^−2^, quick response time of less than 5 s, good stability, selectivity, and reproducibility. Its application for the detection of glucose in human blood serum sample shows acceptable recovery and R.S.D. values. The outstanding glucose sensing performance should be attributed to the unique 3D hierarchical porous superstructure of the composite, especially for its enhanced electron-transfer kinetic properties.

## Introduction

Electrochemical biosensors have been extensively applied to detect biological substances via catalysis and recognition behaviors happening on the surface of electrodes in the fields of medicine, food, industry and environment^1–4^. The generation of electrochemical signal normally includes electrocatalytic reaction happening at the electrolyte/electrode interface, and the electron transfer inside the electrode^5, 6^. Intimate correlation of sensing performance and the structural and electrocatalytic properties of electrodes has motivated great efforts to the design of new materials with superior electrocatalytic activity and electron–transfer kinetics to achieve rapid and sensitive response of electrochemical signal in biosensor^7, 8^.

Metal oxides play an important role in the miniaturization of glucose biosensor due to their inexpensive, good biocompatibility, and excellent electrocatalytic activity along with the controllability of the structure and morphology^9–11^. The effective application of metal oxides is prospective to break through the pivotal limitations of the costly enzymes since the typical glucose oxidase is intrinsically susceptible to the physical and chemical environments^1, 12–14^. Nanostructured metal oxides, such as zero–dimensional (0D) particles, 1D nanowires, 2D nanosheets, and some hollow structures have been widely studied as electrode materials for glucose biosensors with improved sensitivity, reproducibility, and stability. Nickel-based materials, such as NiO and Ni(OH)_2_ have been extensively research as electrocatalyst for glucose due to its redox couple of Ni^3+^/Ni^2+^ in the alkaline medium. However, the poor electronic conductivity of nickel-based materials at room temperature determines the inferior electron–transfer kinetics of the constructed electrodes, which significantly hinders their application in electrochemical biosensors^15–17^. One effective solution to enhance the electron–transfer kinetics in the biosensor is to incorporate a high electrocatalytic activity material with a conductive substance.

Graphene is one of the most popular conductive substance for metal oxides electrode in virtue of its high surface area, excellent electrical conductivity, and superb electrochemical stability^18, 19^. Regrettably, the majority of reported graphene–metal oxide composite materials are composed of nanoparticles, nanoplates or other solid particles, which tend to agglomerate or cumulate on the surface of electrodes^20–22^. Though the resulted low–dimensional agglomerates probably remain large electrode surface area, the electrolyte and the analytes are hampered into the internal space of electrode, the effective electrode/electrolyte interface is extremely reduced^23, 24^. Most of the electrocatalytic reactions merely happen at the external surface of the electrode, the fabricated electrodes exhibit an undesirably inferior electron–transfer kinetics as a consequence of exceptionally long and limited electron–transfer pathway^12, 23, 25^. Recently, 3D porous graphene–based materials have gained much attention for electrochemical energy storage and catalyst support^26^. It is considered to be a new-type material that could afford multidimensional ion–transports and electron–transfer pathways for high–performance electrochemical devices^27–29^. However, the synthesis of 3D porous graphene–metal oxide composite remains to be a daunting challenge and hindered by the complicated and time-consuming template construction and elimination procedures. In addition, an adverse subsequent deposition process of metal oxide markedly obstructs the potential applications of the material in electrochemical biosensors.

In this work, we first propose the fast and facile synthesis of 3D porous NiO hollow sphere/reduced graphene oxide (rGO) composite by applying a 3D Cu_2_O nanosphere/graphene oxide (GO) composite as sacrificial template. The replication and elimination process of the template are accomplished simultaneously within 15 min under mild conditions by masterly controlling the “coordinating etching and precipitating” (CEP) process^30, 31^. The resultant 3D porous NiO hollow sphere/rGO composite is endowed with many advantages for electrochemical applications: firstly, the porous hollow structure of the NiO sphere is conducive to increase the effective surface area and enhance the mass transport kinetics of the electrode; secondly, the 3D rGO skeleton improved the dispersibility and the stability of the NiO hollow spheres, which helps to increase the active sites and the stability of the electrode; more importantly, the direct charge transfer between the NiO and the rGO could generate a more direct and rapid electron-transfer within the electrode material, thereby resulting in an improved electron–transfer kinetics properties of the electrode. As for the application in glucose biosensor, the fabricated electrode based on such composite can detect low-concentration glucose with a high sensitivity of ~2.04 mA·mM^−1^·cm^−2^ and a fast response time of less than 5s. The outstanding glucose-sensing properties are attributed to its unique 3D porous structure, which facilitates superior electrocatalytic activity and remarkable electron-transfer kinetics.

## Results and Discussion

**Figure 1:**
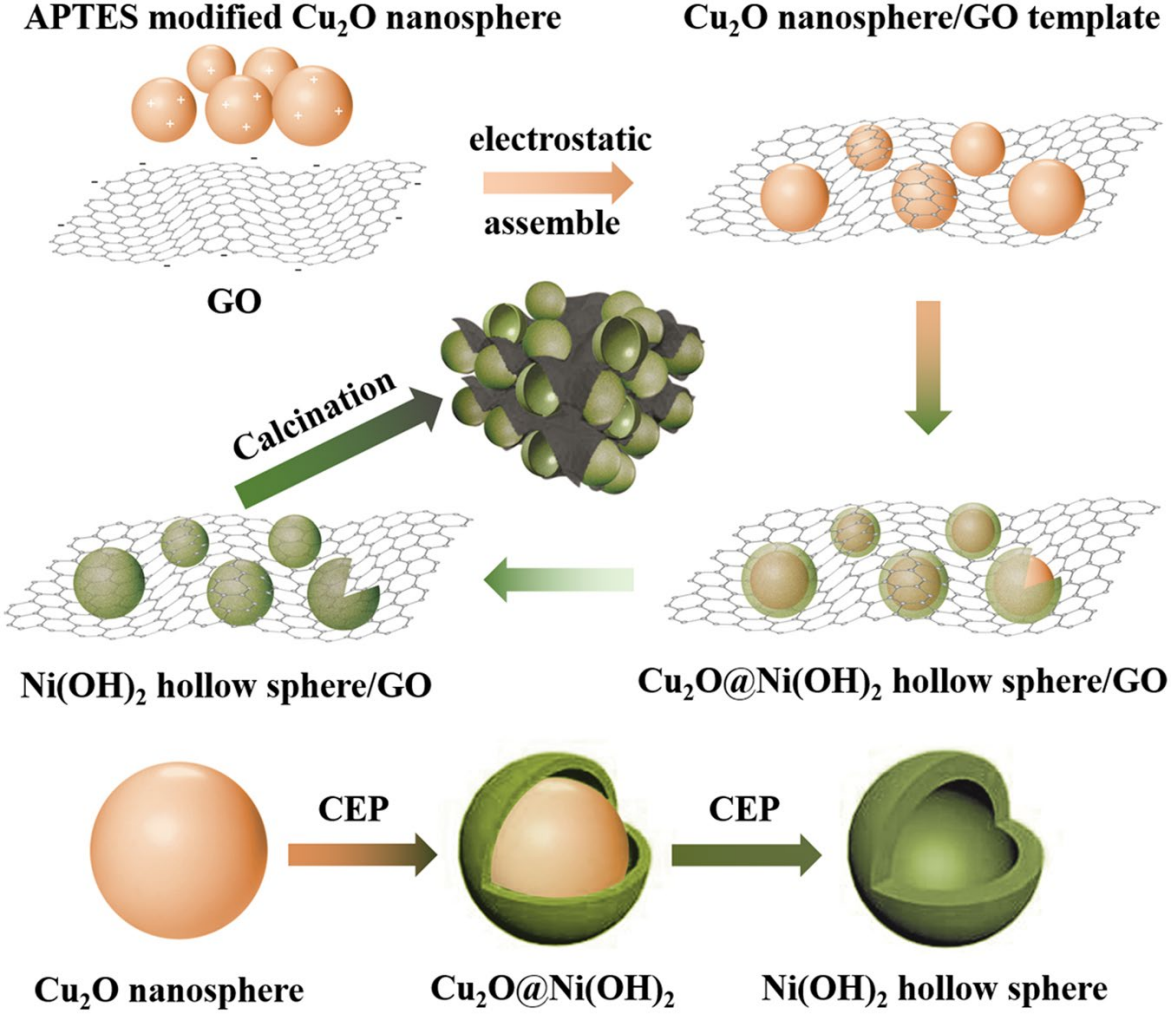
Schematic illustration of the synthesis procedure for 3D NiO hollow sphere/rGO composite. Abbreviations: CEP, coordinating etching and precipitating.

The pre-preparation of the Cu_2_O/GO composite template is very important for the successful preparation of the 3D NiO hollow sphere/rGO composite. Given the factor of energy undulation, graphene is usually in the form of curved surface rather than a plane, and abundant wrinkles are present on the surface. In consideration of the above factors, GO was combined with spherical Cu_2_O with the aim of absorbing more spherical Cu_2_O into GO and more tight contact effect occurring between the materials simultaneously. Figure 1 illustrates the synthesis procedure for 3D NiO hollow sphere/rGO composite. First, the APTES modified positively charged Cu_2_O nanospheres were assembled with the negatively charged GO by electrostatic attraction to obtain the 3D Cu_2_O nanosphere/GO composite. Subsequently, 3D Ni(OH)_2_ hollow sphere/GO composite was rapidly obtained by selecting S_2_O_3_^2−^ as the etchant towards the 3D Cu_2_O/GO composite according to the “CEP” process. Finally, the simple thermal treatment facilitated the formation of 3D NiO hollow sphere/rGO composite. This study proposes for the first time a 3D Cu_2_O nanosphere/GO composite as a hard template for 3D hierarchical porous hybrid material. By subtly controlling the “CEP” process, the replication and the elimination of the template were achieved simultaneously within 15 min under a mild condition. The rapid reaction is attributed to the polycrystalline featured Cu_2_O nanosphere, which supplies plentiful etching interfaces, along with the predesigned premium 3D Cu_2_O/GO composite template with good particle dispersion and mechanical stability.

**Figure 2:**
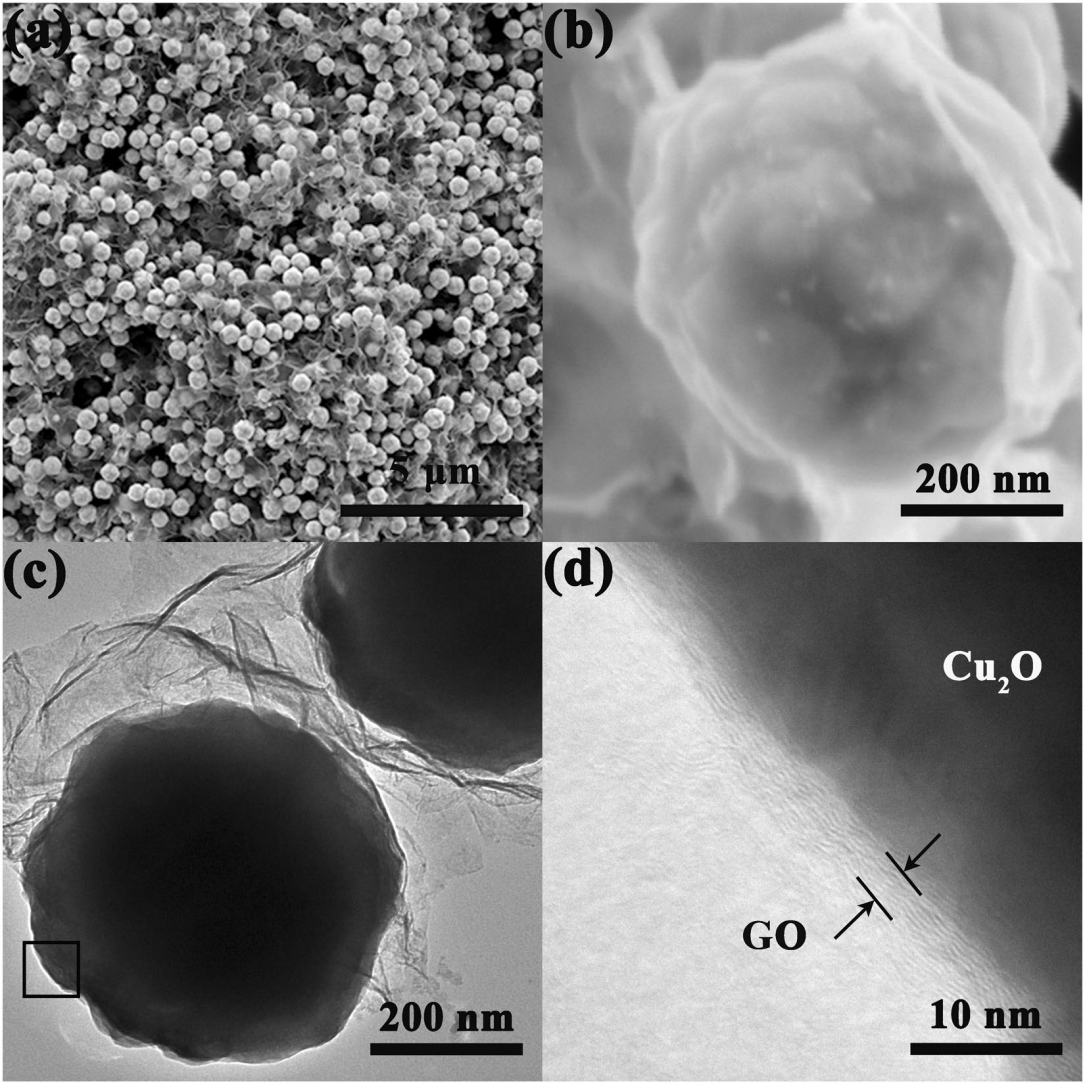
(**a**,**b**) SEM, (**c**) TEM and (**d**) HRTEM images of the Cu_2_O nanosphere/GO composite.

Cu_2_O nanospheres with narrow size distribution were synthesized in our previous work^32^. After modified with a little amount of APTES, the positively charged Cu_2_O nanospheres were assembled with negatively charged GO to obtain a high loading rate of 3D Cu_2_O nanosphere/GO composite. As seen Figure 2a, a large number of Cu_2_O nanospheres with an average size of about 400 nm and rough surface are uniformly distributed in the 3D GO network. High resolution SEM image (Figure 2b) reveals that the solid Cu_2_O nanospheres are actually wrapped in GO nanosheets, and the GO shell displays wrinkled and rough texture because of its flexible and ultrathin features. Spherical Cu_2_O particles were intentionally selected as a stereotype for the assembly of 3D superstructure on account of its isotropic feature that facilitates the order accumulation of the particles from all directions. Moreover, the conspicuous curved surface contact (Figure 2b and c) between Cu_2_O nanospheres and GO nanosheets also contributes to the assembly of 3D superstructure, which not only supplies more effective contact sites at the interface, but also constructs a more flexible and compact stacking. Figure 2d shows the high-resolution TEM image of the selected area marked with a black frame in Figure 2c, which verified that the Cu_2_O nanospheres were closely wrapped by GO nanosheets with thickness of ca. 3 nm.

**Figure 3:**
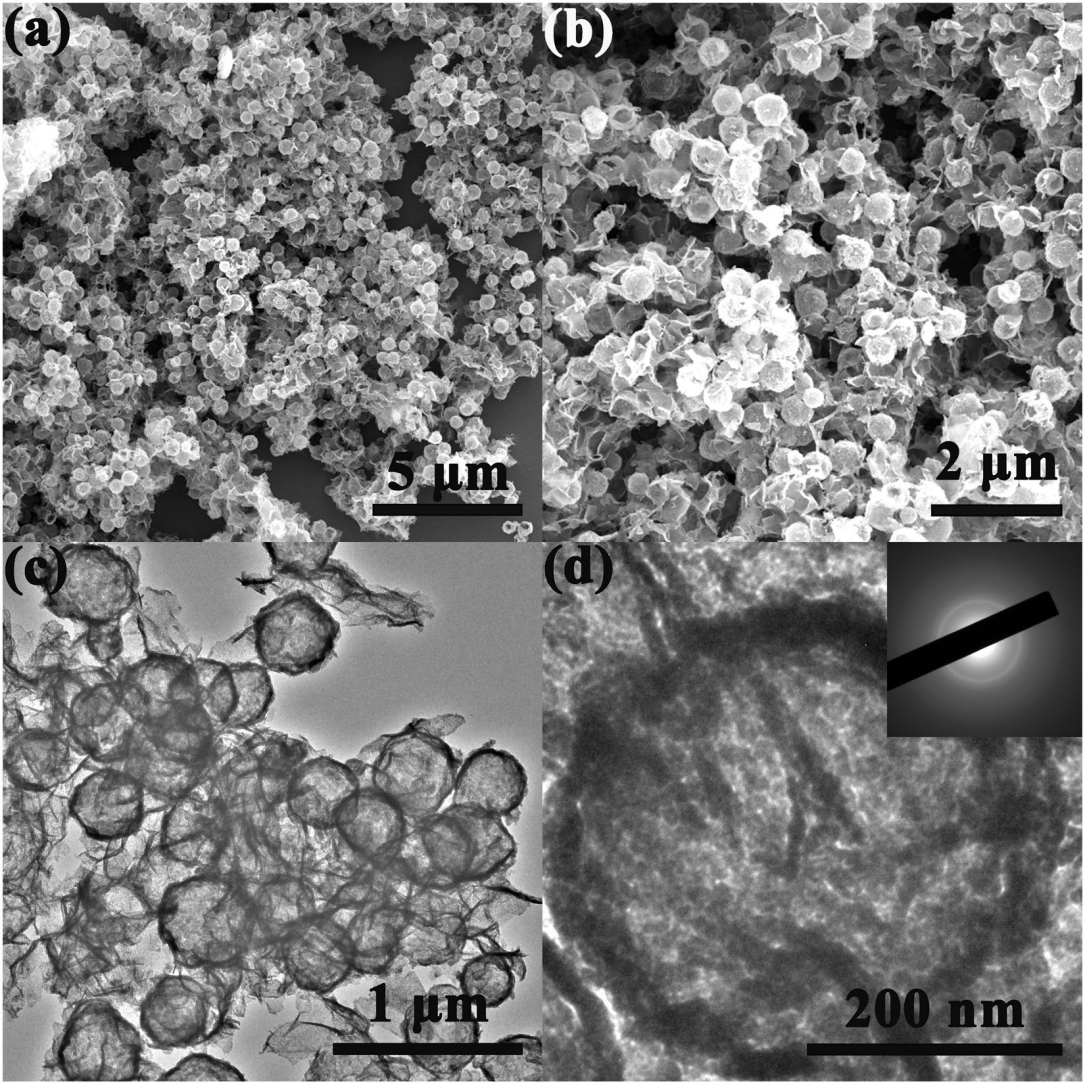
SEM (**a**,**b**) and TEM (**c**,**d**) images of the 3D Ni(OH)_2_ hollow sphere/GO composite. The inset in (**d**) is the corresponding SAED pattern.

By selecting S_2_O_3_^2−^ as the etchant toward the Cu_2_O/GO composite template, 3D Ni(OH)_2_ hollow sphere/GO composite was rapidly synthesized within 15 min in large scale. Figure 3a and b reveal that the Ni(OH)_2_ hollow spheres with an average size of 400 nm well inherited the scale and spherical morphology of Cu_2_O template, and the Ni(OH)_2_ hollow spheres exhibited satisfactory homogeneity and monodispersity in the 3D GO network. The TEM images (Figure 3c and d) uncovered the hollow construction of the Ni(OH)_2_ spherical particles. The inserted SAED pattern in Fig. 3d reveals the amorphous feature of the Ni(OH)_2_ hollow spheres. In this work, the successful synthesis of the 3D Ni(OH)_2_ hollow sphere/GO composite within 15 min should be ascribed to several factors. First, the entire reaction system is controllably under the elaborately designed “CEP” process (illustrated in Figure 1) by adjusting the volume ratio of ethanol and water as well as the concentration of S_2_O_3_^2−^ ^31, 33, 34^. Second, the polycrystalline feature of the Cu_2_O template that has been verified in our previous work is conducive to provide ample interface for the “CEP” process. Compared with the chemical etching time of Guo (about 3 h), the reaction process was substantially cut down to 15 min. Besides, the predesigned 3D Cu_2_O nanosphere/GO composite template with favorable and well dispersion of particles also played a critical role in the controlled synthesis of 3D Ni(OH)_2_ hollow sphere/GO composite.

**Figure 4:**
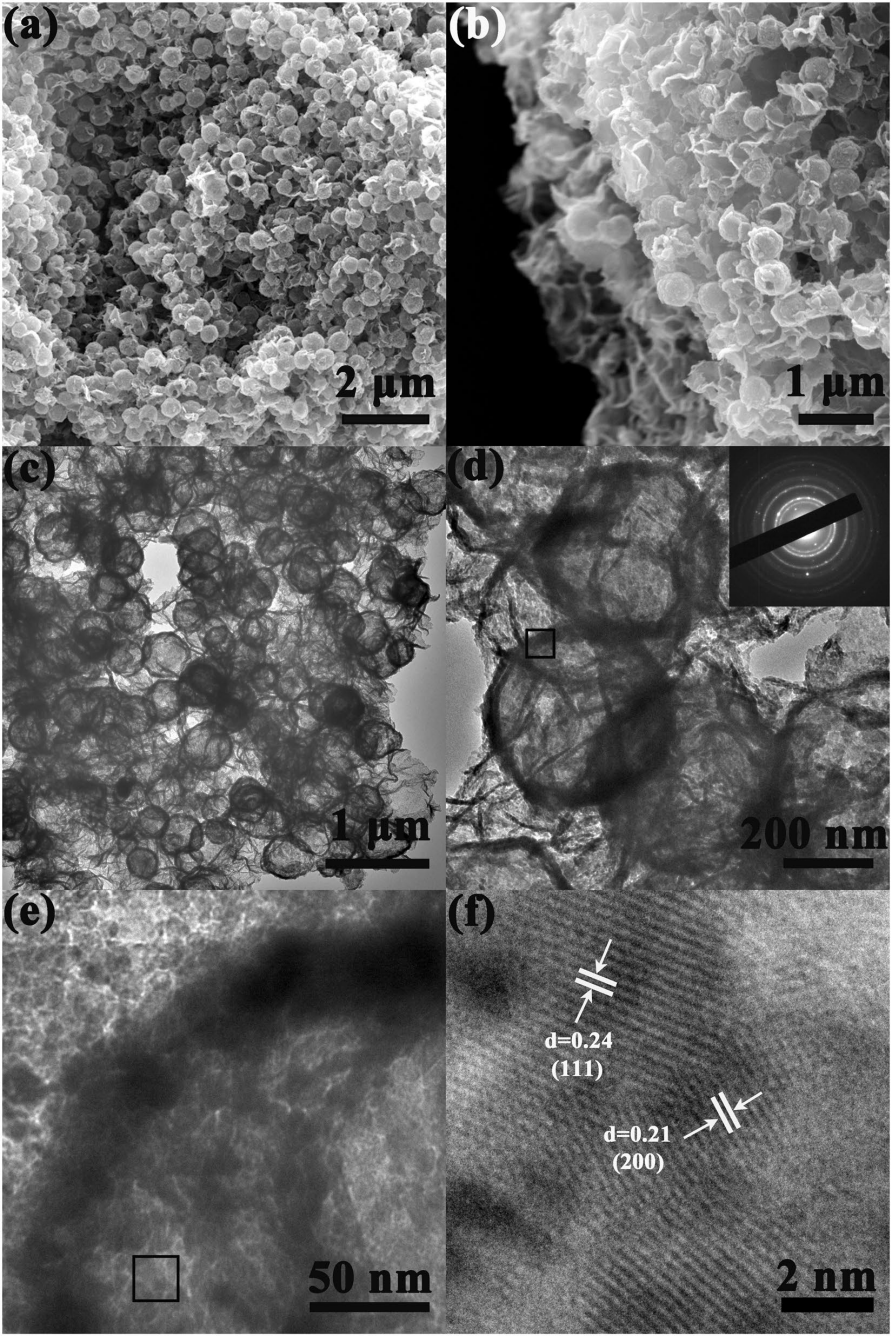
SEM images of 3D NiO hollow sphere/rGO composite (**a**,**b**); TEM images (**c**,**d**,**e**) of NiO hollow sphere/rGO composite, and the inset in (**d**) is the corresponding SAED pattern; ((**f**) HRTEM image of NiO hollow sphere.

After calcination, the as-synthesized Ni(OH)_2_ hollow sphere/GO composite was transformed into NiO hollow sphere/rGO composite, where the latter exhibits more compact 3D stacking than that of the former (Figure 4a), possibly due to the structural shrink of rGO network resulting from the loss of hydrophilic groups. However, the size of hollow spheres well maintained at about 400 nm, suggesting a considerable thermal stability. Figure 4b displays the marginal perspective of the 3D stacking structure, in which the NiO hollow spheres are firmly embedded in the rGO 3D conductive network. More detailed structure information was further characterized by TEM. As seen from Fig. 4c and d, the hollow structured NiO spheres are uniformly distributed in the 3D rGO network, and the surface of the NiO hollow spheres is rough and porous, with a shell thickness of ca. 25 nm (Figure 4e). The inserted SAED pattern in Figure 4d verifies the polycrystalline feature of the NiO hollow spheres. In addition, the HRTEM image in Fig. 4f detects two sets of lattice fringes 0.24 and 0.21 nm, which correspond to the (111) and (200) crystal planes of NiO, respectively. It is worth mentioning that several NiO hollow spheres are fixed in a zigzag by the flexible graphene in Figure 4d. This unique zigzag connection mode not only facilitates the assembly of 3D hierarchical porous superstructure but also helps to increase the contact sites between the NiO and the rGO. The unique 3D hierarchical porous superstructure is endowed with “short cut” mass transport pathways and more efficient surface area, while the increased contact sites are conducive to generate a more direct and rapid electron-transfer within the material.

**Figure 5:**
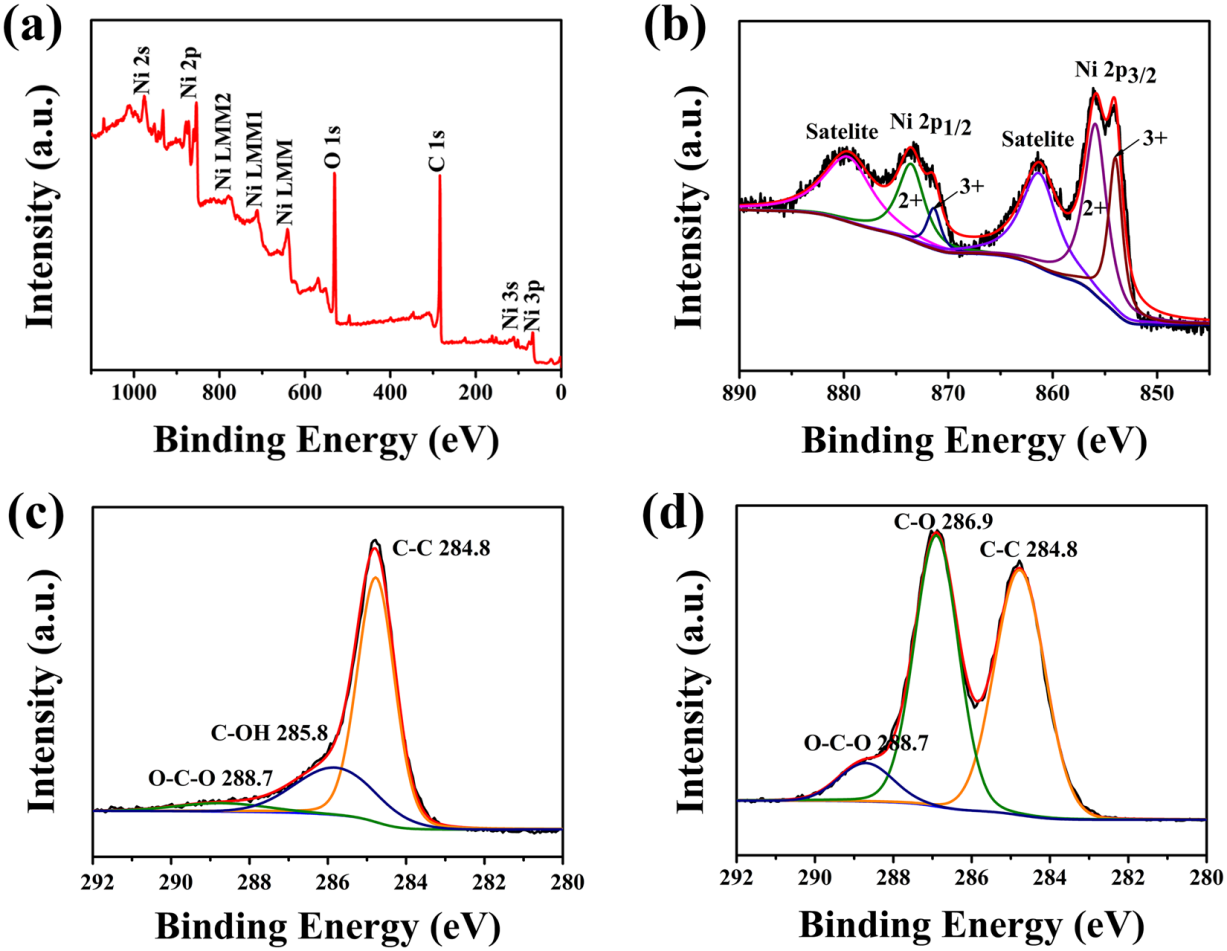
XPS spectra of NiO hollow sphere/rGO composite: (**a**) Survey spectrum, (**b**) Ni 2*p*, (**c**) C 1*s*; (**d**) C 1*s* XPS spectrum of GO.

X-ray photoelectron spectroscopy (XPS) was performed to investigate the detailed elemental information and the oxidation state of the as-prepared NiO hollow sphere/rGO composite. Figure 5a reveals the presence of nickel, oxygen, and carbon. The Ni 2*p* spectrum in Figure 5b displays two edge splits by spin–orbital coupling: the 2*p*_3/2_ main peak at ~854 eV and its satellite at ~862 eV and the 2*p*_1/2_ main peak at ~872 eV and its satellite at ~879 eV, proving the existence of NiO^35, 36^. Figure 5c presents the C 1 *s* spectra of the composite, in which the peaks appearing at 284.8 eV, 285.8 eV, and 288.7 eV coincided with *sp* ^2^ carbon components (C–C bond), C–OH bond derived from the absorbed H_2_O molecule, and C = O double bond components of carboxyl and ketone functions, respectively^37^. Compared with the C 1*s* XPS spectrum of GO in Figure 5d, an evident loss and migration of the oxygen-containing functional groups are observed, suggesting that most of the GO were reduced to rGO by the reduction of S_2_O_3_^2−^ and the thermal treatment^30^. It is conceivable that the high conductive rGO in the composite could function as a 3D conductive network which is conducive to shorten the electron-transfer distance and provide more electron-transfer pathways within the material.

**Figure 6:**
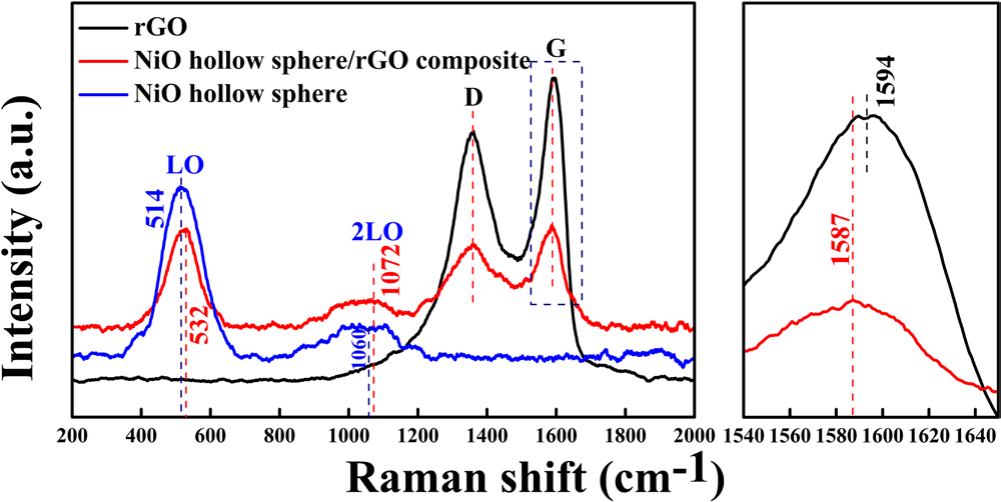
Raman spectra of rGO, NiO hollow sphere, and NiO hollow sphere/rGO composite. Right side shows the enlarged G band of rGO and NiO hollow sphere/rGO composite marked by a rectangle in left.

Raman spectroscopy is powerful tool for the study of carbon materials. Figure 6 shows the Raman spectra of rGO, the NiO hollow sphere, and the NiO hollow sphere/rGO composite. The peaks located at approximately 1358 cm^−1^ and 1587 cm^−1^ for the NiO hollow sphere/rGO composite correspond to the D and G bands of rGO^38^, respectively. It is well known that the G band shift in carbon-based composite is generally related to the charge transfer between the carbon and other compounds^39, 40^. Therefore, the 7 cm^−1^ of band shift from 1594 cm^−1^ to 1587 cm^−1^ of G band should be ascribed to the direct charge transfer between rGO and NiO hollow spheres. Additionally, the peaks located at around 532 and 1072 cm^−1^ belong to the first-order longitudinal optical (LO) and 2LO phonon modes of NiO, respectively^41^. Notably, similar Raman shifts can also be observed for the LO and the 2LO phonon modes, from 514 cm^−1^ to 532 cm^−1^ and from 1060 cm^−1^ to 1070 cm^−1^, respectively. All these results indicate the direct electron-transfer between the 3D rGO conductive network and the NiO hollow spheres, which contributes to reduce the contact resistance and improve the electron-transfer kinetics of the material.

Based on above discussion, it is convincible that the as-prepared 3D porous hybrid material should have advantages in the field of electrochemical, not only for its unique 3D hierarchical porous structural could that provide “short cut” mass transport pathways and more efficient surface area, but also for the distributed 3D rGO conductive network and the direct electron-transfer at the contact sites that contribute to enhance the electron-transfer kinetics of the material. As a typical example, the glucose sensing properties of the 3D NiO hollow sphere/rGO composite was investigated.

**Figure 7:**
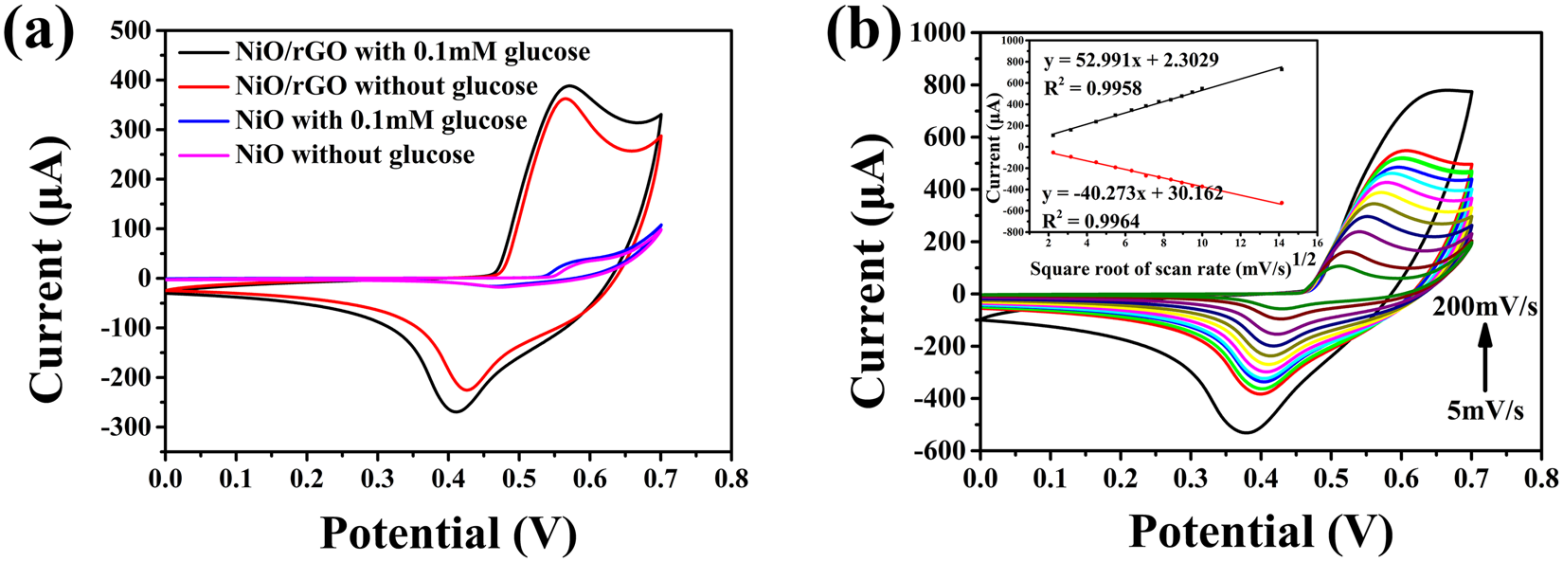
(**a**) CVs of NiO hollow sphere/GCE, NiO hollow sphere/rGO/GCE in 0.1 M NaOH solution in the absence and presence of 0.1 mM glucose. (**b**) CVs of the NiO/rGO/GCE in 0.1 M NaOH in the presence of 0.1 mM glucose at different scan rates. Inset shows the plot of the electrocatalytic current of glucose vs. *v* ^1/2^.

The biosensing performance of the NiO hollow sphere/rGO composite was estimated using a three-electrode system with Ag/AgCl as reference electrode and Pt wire as counter electrode in 0.1 M NaOH electrolyte solution. For comparison, the NiO hollow sphere sample (Figure S1e) was also measured under an identical condition. As shown in Figure 7a, the anodic and cathodic peak potentials positioned at 0.584 V and 0.473 V for the NiO hollow sphere/GCE in absence of glucose can be attributed to the Ni^2+^/Ni^3+^ redox couple in the alkaline medium^42^. However, significantly enhanced redox peaks with anodic and cathodic peak potentials centered at 0.566 V and 0.419 V were observed for the NiO hollow sphere/rGO/GCE. The anodic peak current of the NiO hollow sphere/rGO/GCE (about 0.38 mA) is more than 100-fold higher than that of the NiO hollow sphere/GCE (about 0.03 mA), suggesting a sharply enhanced electrochemical activity of the 3D NiO hollow sphere/rGO composite^17^. This might be attributed to the enhanced electron-transfer kinetics resulting from the 3D rGO conductive network within the composite^14, 43^. As for the anodic peak potential, 0.018 V of negative shift can be observed for the NiO hollow sphere/rGO composite/GCE, indicating an abridged polarization at the surface of the electrode^44^. After the addition of 0.1 mM glucose, apparent current response could be observed for both electrodes, while the rGO/GCE and the bare GCE didn’t show apparent current response of redox peaks (Figure S2). The anodic peaks current differentials (∆*I*) and anodic peak potential differentials (∆*E*) for the NiO hollow sphere/GCE and the NiO hollow sphere/rGO/GCE are 5.96 (∆*I*_1_), 25.42 μA (∆*I*_2_) and 0.002 (∆*E*_1_) and 0.009 V (∆E_2_), respectively. Notably, ∆*I*_2_ > 4∆*I*_2_ and ∆*E*_2_ > 4∆*E*_1_, which confirms a reinforced glucose electrocatalytic capability for the 3D NiO hollow sphere/rGO composite modified electrode. Scanning rate-conducted cyclic voltammograms (CVs) were obtained to further investigate the electrocatalytic process of glucose on the electrode. As shown in Figure 7b, by controlling the scan rate increasing from 5 mV·s^−1^ to 200 mV·s^−1^, the anodic and cathodic peak currents present good linear relationships with the square root of the scan rate with *R* ^2^ values of 0.9958 and 0.9964 (inset in Figure 7b), indicating a typical diffusion-controlled process. The mechanism of glucose oxidation on the electrodes could be described as follows^17, 42, 45^:

1 $${\rm{NiO}}+{{\rm{OH}}}^{-}\to {\rm{NiOOH}}+{{\rm{e}}}^{-}$$

2 $${\rm{NiOOH}}+{\rm{glucose}}\to {\rm{NiO}}+{\rm{glucolactone}}$$

The increase in anodic current is attributed to the electrooxidation of glucose with NiO hollow spheres as an electrocatalyst and accompanied by oxidation from Ni^2+^ to Ni^3+^. The addition and oxidation of glucose would inevitably induce the adsorption of glucose and the oxidized intermediates on active sites of catalyst, and lower the reaction kinetics of electrocatalytic process, thus cause slight positive shift of anodic peak for the electrodes^46, 47^. Even though the electrooxidation of glucose can be expounded reasonably at present, there still need further investigation on elucidating the enhanced electrochemical activity of the NiO hollow sphere/rGO/GCE.

**Figure 8:**
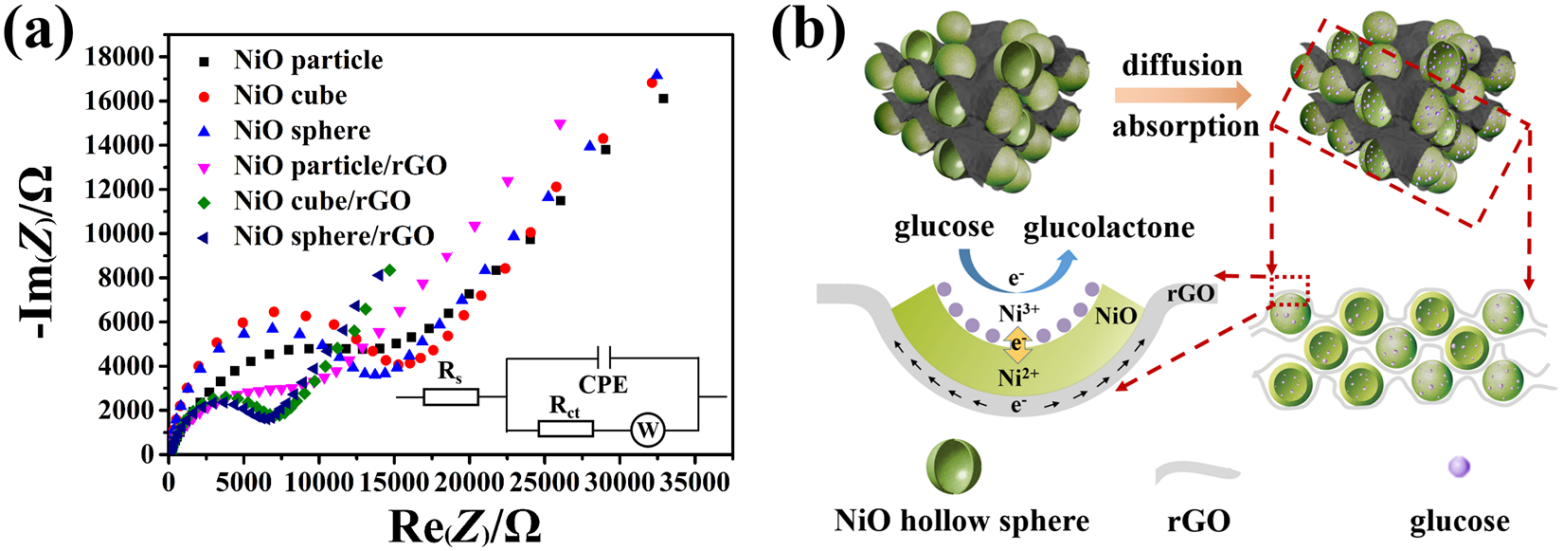
(**a**) Nyquist plots of NiO particle, NiO hollow cube, NiO hollow sphere, NiO particle/rGO, NiO hollow cube/rGO, and NiO hollow sphere/rGO in 0.5 M KCl solution containing 10 mM K_3_Fe(CN)_6_. Inset image illustrates the equivalent circle model of the system. (**b**) Mechanism illustration for the enhanced glucose sensing performance.

Impedance test at a frequency range of 200 kHz to 0.01 Hz was conducted for further understanding of the enhanced electrochemical properties of the NiO hollow sphere/rGO composite. Several samples (the corresponding SEM images are shown in Figure S1) were also measured for comparison. As shown in Figure 8a, on the basis of the equivalent circuit, the electron transfer resistance (*R*_ct_) values at the electrode surface were calculated to be 14788 Ω, 14247 Ω, 14150 Ω, 8183 Ω, 6971 Ω, and 6295 Ω for NiO particle, NiO hollow cube, NiO hollow sphere, NiO particle/rGO, NiO hollow cube/rGO, and NiO hollow sphere/rGO, respectively^48^. The graphene–NiO composites exhibit lower *R*_ct_ than pure NiO materials, and the NiO hollow sphere/rGO composite exhibit significantly decreased *R*_ct_ compared with other materials. It can be concluded that the introducing of rGO conductive network could markedly improve the electron-transfer kinetics of the material, while the construction of 3D porous hollow structure could further enhance the properties by shortening the electron-transfer distance and provide more electron-transfer pathways within the material. Additionally, the NiO hollow sphere (14150 Ω) material exhibits similar *R*_ct_ to that of NiO hollow cube (14247 Ω); by contrast, the NiO hollow sphere/rGO composite (6295 Ω) exhibit evident lower *R*_ct_ than NiO hollow cube/rGO composite (6971 Ω). These results indicate that the combination of NiO hollow spherical and graphene could generate 3D composite materials with better electron transfer kinetics, since the hollow sphere has isotropic feature that could assemble from all directions and create more contact sites. As for the low frequency region, the Warburg impedance (W) of the curves was calculated to be 6431 Ω·S^1/2^, 4462 Ω·S^1/2^, 4445 Ω·S^1/2^, 4469 Ω·S^1/2^, 1995 Ω·S^1/2^, and 1980 Ω·S^1/2^ for NiO particle, NiO hollow cube, NiO hollow sphere, NiO particle/rGO, NiO hollow cube/rGO, and NiO hollow sphere/rGO, respectively^49^. Evidently, the hollow structured materials exhibit much lower W values than those of solid particle materials, indicating improved mass diffusion kinetics of the electrodes^48–50^. This is because electrolyte could sufficiently immerse into the hollow structured electrodes and produce more electrode/electrolyte interface near the GCE, and shorten the electron-transfer distance. Furthermore, as can be seen from Figure 7, with similar mass diffusion kinetics properties, the NiO hollow sphere/rGO/GCE exhibits much stronger response current toward glucose to that of the NiO hollow sphere/GCE, and that the kinetic process of glucose oxidation at the NiO hollow sphere/rGO/GCE is diffusion-controlled, which also indirectly proved that the significantly enhanced electrochemical activity was mainly derived from the excellent electron-transfer kinetics of the 3D NiO hollow sphere/rGO material^43^. Figure 8b illustrated the mechanism for the enhanced glucose sensing performance of the 3D NiO hollow sphere/rGO composite, which can be summarized as the following points: first, the porous hollow structure of the NiO sphere could provide more active sites and diffusion pathways for adsorbing more glucose; second, the 3D rGO conductive network improved the dispersibility of the NiO hollow spheres, and that enhanced the electron-transfer kinetics of the composite; third, the direct charge transfer between the NiO and the rGO could generate a more direct and rapid electron-transfer at the interface, and resulting an improved electron–transfer kinetics properties.

**Figure 9:**
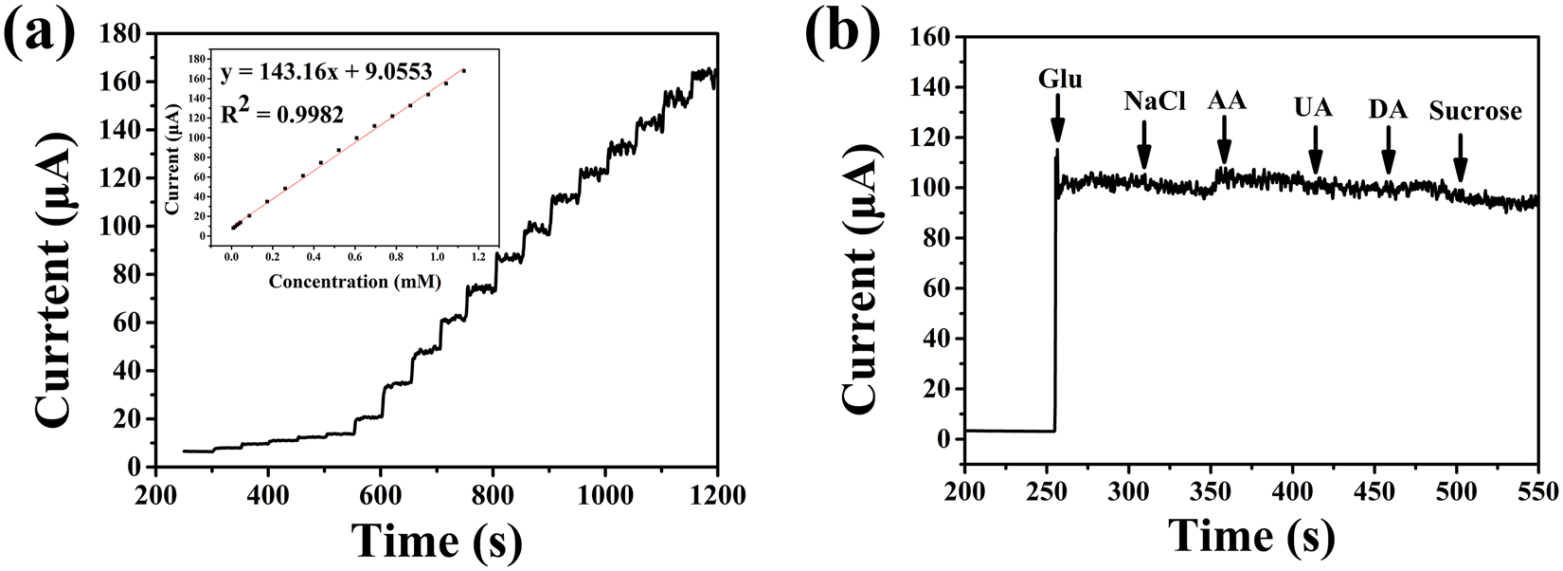
(**a**) Amperometric response of NiO hollow sphere/rGO/GCE at 0.58 V with the successive addition of glucose in 0.1 M NaOH. Inset shows the corresponding calibration curve. (**b**) Interference test of NiO hollow sphere/rGO/GCE toward the addition of glucose (Glu, 0.6 mM) and various interferents, including NaCl (0.06 mM), ascorbic acid (AA, 0.06 mM), uric acid (UA, 0.06 mM), dopamine (DA, 0.1 mM), sucrose (Suc, 0.06 mM), urea (0.06 mM), and cholesterol (Chol, 0.06 mM) in 0.1 M NaOH.

After successive stepwise addition of glucose into 0.1 M NaOH at a working potential of 0.58 V, the NiO hollow sphere/rGO/GCE shows a good linear range from 0.009 mM to 1.129 mM with a correlation coefficient of 0.998 and a slope of 143.16 in Figure 9a. The sensitivity of the NiO hollow sphere/rGO/GCE is calculated to be 2.04 mA mM^−1^ cm^−2^, and the limit of detection is as low as 82 nM (*S/N* = 3). Compared with most of other nickel-based or composite materials **(**Table S1 **)**, the NiO hollow sphere/rGO/GCE exhibits a higher sensitivity and larger linear range in glucose detection. The excellent glucose sensing performance again reveals that the as-prepared 3D NiO hollow sphere/rGO composite is a novel and promising electrocatalytic material that allows for efficient electron-transfer, consequently generating rapid response and enhanced sensitivity even at a low detection concentration.

Then, interference study was carried out by introducing ascorbic acid, uric acid, and dopamine as interferential compounds. Considering that the concentration of glucose in human blood is considerably higher (by an order of magnitude at minimum) than that of the other interferential species, the interference experiment was conducted by the addition of 0.6 mM glucose and successive addition of 0.06 mM other interferential species in 0.1 M NaOH. As shown in Figure 9b, the negligible current enhancement caused by the addition of other interferential species clearly demonstrates that the NiO hollow sphere/rGO composite-modified electrode exhibit excellent selectivity towards glucose.

Stability and reproducibility experiments were also conducted to evaluate the performance of the electrode. Five successive amperometric measurements of glucose on the same electrode yield a reproducible current with the low relative standard deviation (R.S.D.) of 1.50%, demonstrating superior reproducibility. The storage stability of the electrode stored at 4 °C was investigated for seven days by testing the response current to the same concentration of glucose solution. The retained 94.9% of the initial response revealed that the electrode possesses good long-term stability. In human blood serum samples with different concentrations of glucose, the as-prepared electrode exhibited acceptable recovery and R.S.D. values (Table S2).

## Conclusion

In summary, the 3D NiO hollow sphere/rGO composite was successfully synthesized by chemical etching toward the Cu_2_O nanosphere/GO template according to the “CEP” process. The NiO hollow spheres were uniformly embedded in the 3D rGO conductive network and formed a unique 3D hierarchical porous superstructure with superior mass-transport and electron-transfer kinetics properties. Serving as electrode material, the 3D NiO hollow sphere/rGO composite exhibits excellent glucose sensing performance with high sensitivity of 2.04 mA·mM^−1^·cm^−2^ and wide linear range from 0.009 mM to 1.129 mM. The outstanding glucose sensing performance should be attributed to the unique 3D hierarchical porous superstructure of the composite, especially for its enhanced electron-transfer kinetic properties. Except for glucose-sensing, the as-prepared 3D NiO hollow sphere/rGO composite can also be useful in many other fields such as supercapacitor, lithium ion battery, and electrocatalysis.

## Methods

### Synthesis of Cu_2_O nanosphere/GO composite

First, Cu_2_O nanospheres were synthesized according to our previous work^32^. 0.1 g of the as-prepared Cu_2_O nanospheres were ultrasonically dispersed into 9.9 mL of isopropanol, followed by adding 0.1 mL of 3-aminopropyltriethoxysilane (APTES) and being stirred at room temperature for 24 h. After being washed with ethanol repeatedly, the APTES-modified Cu_2_O nanospheres were collected and dried at 60 °C overnight. For obtaining Cu_2_O nanosphere/GO composite, 0.1 g of APTES-modified Cu_2_O nanospheres were ultrasonically dispersed into 30 mL of deionized water, followed by adding 20 mL of GO aqueous solution (1 mg·mL^−1^) and then being stirred at room temperature for 2 h. Finally, the Cu_2_O nanosphere/GO composite template was collected by centrifugation and washed with deionized water.

### Synthesis of NiO hollow sphere/rGO composite

10 mg of Cu_2_O nanosphere/GO composite, 1.7 mg of NiCl_2_·6H_2_O and 0.33 g of PVP (K30, MW≈3800) were dispersed into a mixed solvent of ethanol and deionized water (*V*_ethanol_:*V*_water_ = 1:1, 10 mL). After being stirred for 10 min, 4 mL of Na_2_S_2_O_3_ aqueous solution (0.8 M) was added dropwise into the mixture for the “coordinating etching and precipitating” (CEP). Approximately 15 min later, the color of the solution gradually became stable, the solid products (Ni(OH)_2_ hollow sphere/GO composite) were collected after several rinse–centrifugation cycles with deionized water and ethanol. Finally, the Ni(OH)_2_ hollow sphere/GO composite was heated to 350 °C at a ramp rate of 1 °C min^−1^ in a horizontal tube furnace under an Ar environment and kept at this temperature for 4 h to obtain NiO hollow sphere/rGO composite. The synthesis of rGO, NiO particle, NiO hollow sphere, and NiO particle/rGO composite were similar to those of NiO hollow sphere/rGO composite, expect for the absence of NiCl_2_·6H_2_O, Cu_2_O nanosphere or GO, respectively. By replacing Cu_2_O nanosphere with Cu_2_O nanocube referred to Guo’s report^51^. NiO hollow cube and NiO hollow cube/rGO composite were also obtained.

### Materials characterization

The morphologies of the samples were characterized using a Hitachi S–4800 field–emission scanning electron microscope (FE–SEM). The X–ray photoelectron spectroscopy (XPS) data were obtained with Thermo Scientific ESCALAB250 using Al radiation. Transmission electron microscopy (TEM) micrographs were acquired using a JEOL–2100 F microscope. The Raman spectrum of the samples was procured via Reflex Laser Raman spectrograph (Renishaw Corporation). Electrochemical characterizations were performed using the electrochemical workstation SP–200 (Bio–Logic science Instruments). Pt wire and Ag/AgCl (saturated KCl) were used as counter and reference electrodes, respectively.

### Electrochemical measurements

The glassy carbon electrode (GCE) was polished to a smooth surface with 0.05 μm alumina powders. The electrode was sonicated successively in ethanol and deionized water for 5 min, respectively. The aqueous solution of the samples were added dropwise onto the GCE surface and air–dried at room temperature.

## Electronic supplementary material

3D NiO hollow sphere/reduced graphene oxide composite for high-performance glucose biosensor

## References

[1] Ronkainen NJ, Halsall HB, Heineman WR (2010). Electrochemical biosensors. Chemical Society Reviews.

[2] Wang J (2008). Electrochemical glucose biosensors. Chemical reviews.

[3] Im H, Huang XJ, Gu B, Choi YK (2007). A dielectric-modulated field-effect transistor for biosensing. Nat Nano.

[4] Xiang Y, Lu Y (2011). Using personal glucose meters and functional DNA sensors to quantify a variety of analytical targets. Nat Chem.

[5] Minteer SD, Atanassov P, Luckarift HR, Johnson GR (2012). New materials for biological fuel cells. Materials Today.

[6] Kimmel DW, LeBlanc G, Meschievitz ME, Cliffel DE (2011). Electrochemical sensors and biosensors. Analytical Chemistry.

[7] Yang W (2010). Carbon Nanomaterials in Biosensors: Should You Use Nanotubes or Graphene?. Angewandte Chemie International Edition.

[8] Lang, X.-Y. *et al*. Nanoporous gold supported cobalt oxide microelectrodes as high-performance electrochemical biosensors. *Nat Commun* **4** (2013). 10.1038/ncomms3169 23851924

[9] Solanki PR, Kaushik A, Agrawal VV, Malhotra BD (2011). Nanostructured metal oxide-based biosensors. NPG Asia Mater.

[10] Lou XW, Archer LA, Yang Z (2008). Hollow Micro-/Nanostructures: Synthesis and Applications. Advanced Materials.

[11] Lai X, Halpert JE, Wang D (2012). Recent advances in micro-/nano-structured hollow spheres for energy applications: From simple to complex systems. Energy & Environmental Science.

[12] Dong X-C (2012). 3D Graphene–Cobalt Oxide Electrode for High-Performance Supercapacitor and Enzymeless Glucose Detection. ACS Nano.

[13] Yu Z (2016). Facile synthesis of NiCo_2_O_4_@Polyaniline core–shell nanocomposite for sensitive determination of glucose. Biosensors and Bioelectronics.

[14] Li SJ (2014). A facile one-step electrochemical synthesis of graphene/NiO nanocomposites as efficient electrocatalyst for glucose and methanol. Sensors and Actuators B: Chemical.

[15] Feinleib J, Adler D (1968). Band Structure and Electrical Conductivity of NiO. Physical Review Letters.

[16] Zhang WD, Chen J, Jiang LC, Yu YX, Zhang JQ (2010). A highly sensitive nonenzymatic glucose sensor based on NiO-modified multi-walled carbon nanotubes. Microchimica Acta.

[17] Zhang Y, Wang Y, Jia J, Wang J (2012). Nonenzymatic glucose sensor based on graphene oxide and electrospun NiO nanofibers. Sensors and Actuators B: Chemical.

[18] Dreyer DR, Park S, Bielawski CW, Ruoff RS (2010). The chemistry of graphene oxide. Chemical Society Reviews.

[19] Novoselov KS (2012). A roadmap for graphene. Nature.

[20] Wu ZS (2012). Graphene/metal oxide composite electrode materials for energy storage. Nano Energy.

[21] Srivastava M (2015). Recent advances in graphene and its metal-oxide hybrid nanostructures for lithium-ion batteries. Nanoscale.

[22] Li Q, Mahmood N, Zhu J, Hou Y, Sun S (2014). Graphene and its composites with nanoparticles for electrochemical energy applications. Nano Today.

[23] Xia XH, Chao DL, Zhang YQ, Shen ZX, Fan HJ (2014). Three-dimensional graphene and their integrated electrodes. Nano Today.

[24] He Y (2013). Freestanding Three-Dimensional Graphene/MnO_2_ Composite Networks As Ultralight and Flexible Supercapacitor Electrodes. ACS Nano.

[25] Dong X (2012). 3D Graphene Foam as a Monolithic and Macroporous Carbon Electrode for Electrochemical Sensing. ACS Applied Materials & Interfaces.

[26] Shi Q (2016). 3D graphene-based hybrid materials: synthesis and applications in energy storage and conversion. Nanoscale.

[27] Chen CM (2012). Macroporous ‘bubble’ graphene film via template-directed ordered-assembly for high rate supercapacitors. Chemical Communications.

[28] Wang ZL, Xu D, Wang HG, Wu Z, Zhang XB (2013). *In Situ* Fabrication of Porous Graphene Electrodes for High-Performance Energy Storage. ACS Nano.

[29] Choi BG, Yang M, Hong WH, Choi JW, Huh YS (2012). 3D Macroporous Graphene Frameworks for Supercapacitors with High Energy and Power Densities. ACS Nano.

[30] Guan X (2014). CoO Hollow Cube/Reduced Graphene Oxide Composites with Enhanced Lithium Storage Capability. Chemistry of Materials.

[31] Nai J, Tian Y, Guan X, Guo L (2013). Pearson’s Principle Inspired Generalized Strategy for the Fabrication of Metal Hydroxide and Oxide Nanocages. Journal of the American Chemical Society.

[32] Xu, C. F. *et al*. Fast Synthesis of Hierarchical Co(OH)_2_ Nanosheet Hollow Spheres with Enhanced Glucose Sensing. *European Journal of Inorganic Chemistry* 3163–3168 (2016).

[33] Nai J, Wang S, Bai Y, Guo L (2013). Amorphous Ni(OH)_2_ Nanoboxes: Fast Fabrication and Enhanced Sensing for Glucose. Small.

[34] Wang Z, Luan D, Boey FYC, Lou XW (2011). Fast Formation of SnO_2_ Nanoboxes with Enhanced Lithium Storage Capability. Journal of the American Chemical Society.

[35] Zhou G (2012). Oxygen Bridges between NiO Nanosheets and Graphene for Improvement of Lithium Storage. ACS Nano.

[36] Wu C (2014). Preparation of Novel Three-Dimensional NiO/Ultrathin Derived Graphene Hybrid for Supercapacitor Applications. ACS Applied Materials & Interfaces.

[37] Cai G-f (2012). An efficient route to a porous NiO/reduced graphene oxide hybrid film with highly improved electrochromic properties. Nanoscale.

[38] Ferrari AC, Robertson J (2000). Interpretation of Raman spectra of disordered and amorphous carbon. Physical Review B.

[39] Rao AM, Eklund PC, Bandow S, Thess A, Smalley RE (1997). Evidence for charge transfer in doped carbon nanotube bundles from Raman scattering. Nature.

[40] Zhang H (2015). NiCo_2_O_4_/N-doped graphene as an advanced electrocatalyst for oxygen reduction reaction. Journal of Power Sources.

[41] Mironova-Ulmane N (2007). Raman scattering in nanosized nickel oxide NiO. Journal of Physics: Conference Series.

[42] Ci S (2014). Nickel oxide hollow microsphere for non-enzyme glucose detection. Biosensors and Bioelectronics.

[43] Liu C, Wang K, Luo S, Tang Y, Chen L (2011). Direct Electrodeposition of Graphene Enabling the One-Step Synthesis of Graphene–Metal Nanocomposite Films. Small.

[44] Kang X (2010). A graphene-based electrochemical sensor for sensitive detection of paracetamol. Talanta.

[45] Dung NQ, Patil D, Jung H, Kim J, Kim D (2013). NiO-decorated single-walled carbon nanotubes for high-performance nonenzymatic glucose sensing. Sensors and Actuators B: Chemical.

[46] Lu P (2015). Synthesis and characterization of nickel oxide hollow spheres–reduced graphene oxide–nafion composite and its biosensing for glucose. Sensors and Actuators B: Chemical.

[47] Zheng L, Zhang J (2009). q. & Song, J. f. Ni(II)–quercetin complex modified multiwall carbon nanotube ionic liquid paste electrode and its electrocatalytic activity toward the oxidation of glucose. Electrochimica Acta.

[48] Rubinstein I, Rishpon J, Gottesfeld S (1986). An AC‐Impedance Study of Electrochemical Processes at Nafion‐Coated Electrodes. Journal of The Electrochemical Society.

[49] Yang D (2016). Electrochemical Impedance Studies of CO_2_ Reduction in Ionic Liquid/Organic Solvent Electrolyte on Au Electrode. Electrochimica Acta.

[50] Kim DJ (2013). Diffusion behavior of sodium ions in Na_0.44_MnO_2_ in aqueous and non-aqueous electrolytes. Journal of Power Sources.

[51] Zhang DF (2009). Delicate control of crystallographic facet-oriented Cu_2_O nanocrystals and the correlated adsorption ability. Journal of Materials Chemistry.

