# Supplementary material for: 3D NiO hollow sphere/reduced graphene oxide composite for high-performance glucose biosensor
Source: Sci Rep. 2017 Jul 12;7:5220. doi: 10.1038/s41598-017-05528-1 (PMC5507916; doi:10.1038/s41598-017-05528-1)
Supplement: Supplementary file 1 — 3D NiO hollow sphere/reduced graphene oxide composite for high-performance glucose biosensor [file 41598_2017_5528_MOESM1_ESM.doc]

**Supplementary Information**

**3D NiO hollow sphere/reduced graphene oxide composite for high-performance glucose biosensor**

Wei Huang^a^, Shujiang Ding^b^, Yong Chen^a^, Wanjun Hao^a^, Xiaoyong Lai^c^, Juan Peng^c^, Jinchun Tua[[1]](#footnote-2)*, Yang Caoa[[2]](#footnote-3)*, Xiaotian Li^d^

^a^ *State Key Laboratory of Marine Resource Utilization in South China Sea, Key Laboratory of Tropical Biological Resources of Ministry of Education Hainan University, Haikou 570228, P. R. China*

^b^ *Department of Applied Chemistry, School of Science, State Key Laboratory for Mechanical Behavior of Materials and MOE Key Laboratoryfor Nonequilibrium Synthesis and Modulation of Condensed Matter, Xi’an Jiaotong University, Xi’an 710049, P. R. China*

^c^ *Laboratory Cultivation Base of Natural Gas Conversion, School of Chemistry and Chemical Engineering, Ningxia University, Yinchuan 750021, P. R. China.*

^d^ *Key Laboratory of Automobile Materials of Ministry of Education, School of Material Science and Engineering, Jilin University, Changchun 130012, P.R. China.*


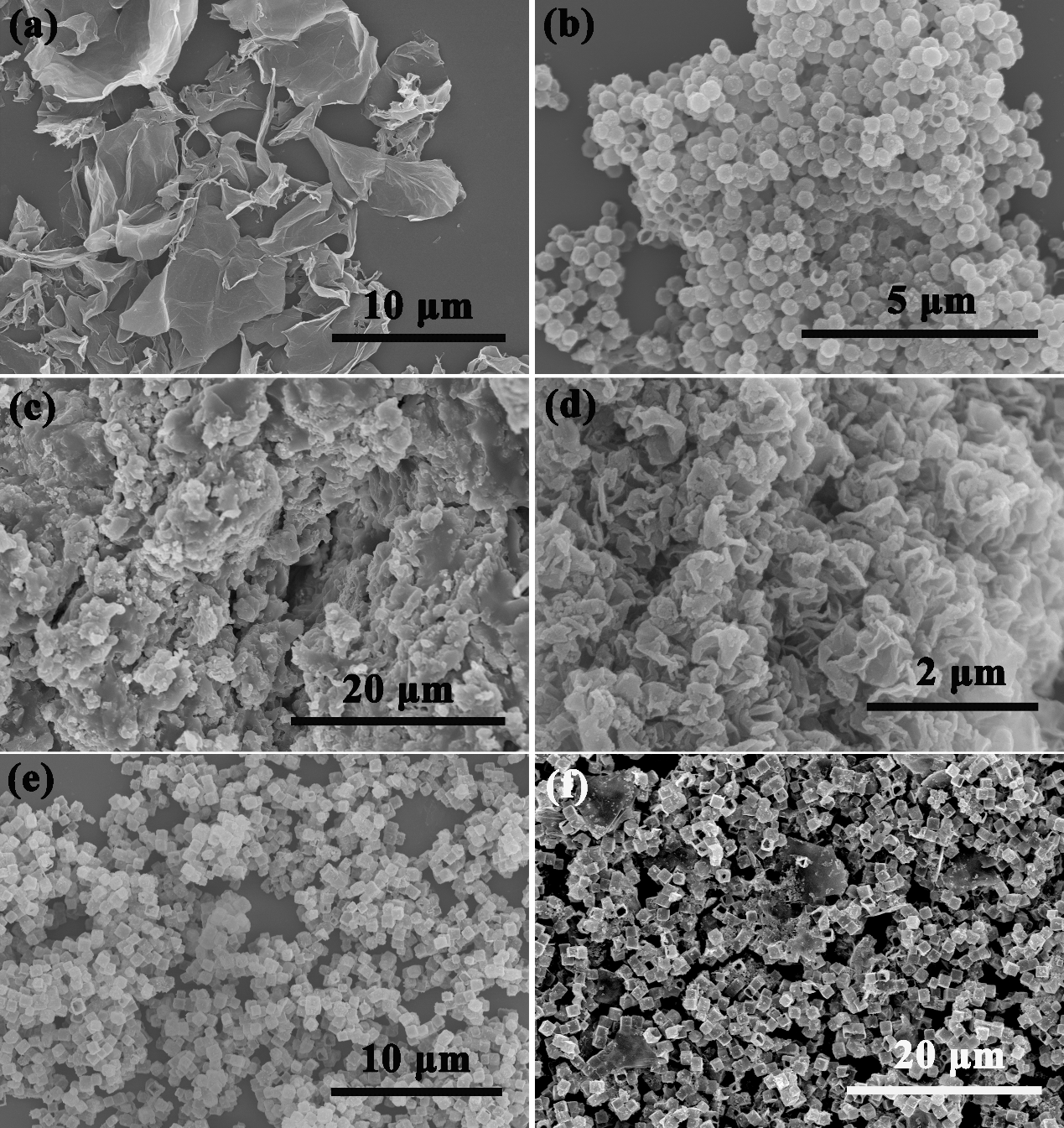


**Fig. S1** SEM images of (a) rGO, (b) NiO hollow sphere, (c) NiO particle, (d) NiO particle/rGO composite, (e) NiO hollow cube, and (f) NiO hollow cube/rGO composite.


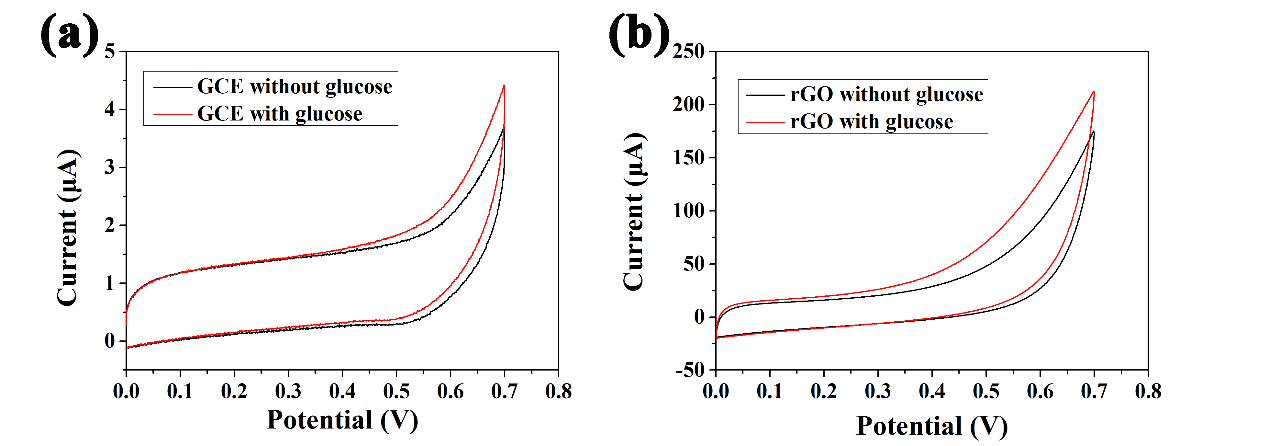


**Fig. S2** CVs of (a) bare GCE and (b) rGO/GCE in 0.1 M NaOH solution in the absence and presence of 0.1 mM glucose.

**Table S1** Comparison of the 3D NiO hollow sphere/rGO composite/GCE with other Ni-based nonenzymatic glucose sensors.

| Type of electrode | linear range  (μM) | Detection limit (μM) | Sensitivity (μA·mM^-1^·cm^-2^) | Reference |
| --- | --- | --- | --- | --- |
| rGO/NiO | 9 - 1129 | 0.082 | 2040 | Present work |
| Ni-rGO | 1-110 | 1 | 813 | 1 |
| Ni(OH)_2_/CNFs | 1-1200 | 0.76 | 1038.6 | 2 |
| NiO/Pt/ERGO | 50-5660 | 0.2 | 668.2 | 3 |
| porous NiO thin films | 0 -1000 | 0.34 | 1680 | 4 |
| NiO/FTO | 10 - 800 | 1.2 | 8500 | 5 |
| Graphene oxide/nickel oxide | 3.13 - 3050 | 1 | 1087 | 6 |
| amorphous Ni(OH)_2_ nanoboxes | 0.5-500 | 0.07 | 487.3 | 7 |
| NiO hollow nanospheres | 1500 - 7000 | 47 | 343 | 8 |
| NiO/CNTs | 200 - 12000 | 160 | 436 | 9 |

CNFs: carbon nanofibers

GNs: graphene nanosheets

CNTs: carbon nanotubes

**Table S2** The testing results of the real samples with different glucose concentrations on the 3D NiO hollow sphere/rGO composite/GCE (N=3).

| Concentration of glucose in human blood serum  (mM) | Concentration obtained with our method  (mM)^a^ | Recovery (%) | Relative standard deviation (RSD) (%)^b^ |
| --- | --- | --- | --- |
| 4.4 | 4.30 | 97.7 | 2.55 |
| 3.0 | 2.96 | 98.7 | 2.47 |
| 2.0 | 1.97 | 98.5 | 2.32 |
| 1.0 | 0.98 | 98 | 2.14 |
| 0.5 | 0.51 | 102 | 2.01 |

^a^ The average value of three parallel experiments

^b^ Calculated from the three parallel experiments

**References**

1 Wang, Z., Hu, Y., Yang, W., Zhou, M. & Hu, X. Facile one-step microwave-assisted route towards Ni nanospheres/reduced graphene oxide hybrids for non-enzymatic glucose sensing. *Sensors* **12**, 4860-4869 (2012).

2 Zhang, L., Yuan, S.-m. & Lu, X.-j. Amperometric nonenzymatic glucose sensor based on a glassy carbon electrode modified with a nanocomposite made from nickel(II) hydroxide nanoplates and carbon nanofibers. *Microchimica Acta* **181**, 365-372 (2014).

3 Li, M., Bo, X., Mu, Z., Zhang, Y. & Guo, L. Electrodeposition of nickel oxide and platinum nanoparticles on electrochemically reduced graphene oxide film as a nonenzymatic glucose sensor. *Sensors and Actuators B: Chemical* **192**, 261-268 (2014).

4 Garcia-Garcia, F. J., Salazar, P., Yubero, F. & González-Elipe, A. R. Non-enzymatic Glucose electrochemical sensor made of porous NiO thin films prepared by reactive magnetron sputtering at oblique angles. *Electrochimica Acta* **201**, 38-44 (2016).

5 Wang, G. *et al.* Free-standing nickel oxide nanoflake arrays: synthesis and application for highly sensitive non-enzymatic glucose sensors. *Nanoscale* **4**, 3123-3127 (2012).

6 Yuan, B. *et al.* Graphene oxide/nickel oxide modified glassy carbon electrode for supercapacitor and nonenzymatic glucose sensor. *Electrochimica Acta* **88**, 708-712 (2013).

7 Nai, J., Wang, S., Bai, Y. & Guo, L. Amorphous Ni(OH)_2_ Nanoboxes: Fast Fabrication and Enhanced Sensing for Glucose. *Small* **9**, 3147-3152 (2013).

8 Li, C. *et al.* A novel amperometric biosensor based on NiO hollow nanospheres for biosensing glucose. *Talanta* **77**, 455-459 (2008).

9 Shamsipur, M., Najafi, M. & Hosseini, M.-R. M. Highly improved electrooxidation of glucose at a nickel(II) oxide/multi-walled carbon nanotube modified glassy carbon electrode. *Bioelectrochemistry* **77**, 120-124 (2010).

1. ^*^ Corresponding author. Tel: +86 898 66259764; fax: +86 898 66259764.

   *E–mail address*: tujinchun@hainu.edu.cn [↑](#footnote-ref-2)
2. ^*^ Corresponding author. Tel: +86 898 66259764; fax: +86 898 66259764.

   *E–mail address*: cy507@hainu.edu.cn [↑](#footnote-ref-3)
